# A palliative care rapid access clinic reduces emergency department visits: a retrospective single centre analysis

**DOI:** 10.1186/s12904-025-01833-z

**Published:** 2025-07-08

**Authors:** Wendy Kinton, Timothy Roberts, Maureen Mitchell, Nicolas Smoll, Marco Giuseppin

**Affiliations:** 1 University of the Sunshine Coast, 90 Sippy Downs Drive, Sippy Downs, QLD 4556 Australia; 2 Sunshine Coast Health, 6 Doherty St, Birtinya, QLD 4575 Australia; 3 Sunshine Coast Public Health Unit, PO Box 577, Maroochydore, QLD 4558 Australia

**Keywords:** Emergency department visit, Mortality, Palliative care, Rapid access, Symptom management

## Abstract

**Background:**

Patients with palliative care needs often rely on emergency departments for management of acute symptoms due to limited access to timely and appropriate outpatient care, however they can be poorly equipped to meet patients’ complex needs. Rapid access clinics exist for addressing health issues such as chest pain but are not routinely established for palliative care. In 2020, the Sunshine Coast Health Palliative Care Service introduced a rapid access clinic to address patients’ unmet acute care needs. This research aimed to understand the impact on clinical outcomes.

**Methods:**

A retrospective observational analysis of patient health records was undertaken for 283 admissions for 172 patients who attended the clinic between 1 January 2020 and 31 December 2022, and included demographic and diagnostic information, reason for admission and date of death. Statistical analysis of differences using the chi squared test was conducted for age (< 70 years vs. ≥ 70 years), gender and mortality at 30 days after discharge from the clinic. Fisher’s exact test was used to assess associations between the type of admission and the likelihood of preventing an emergency department visit. Confidence interval was set at 95%.

**Results:**

Attendance at the rapid access clinic was judged to likely result in avoidance of an emergency department visit for 11.7% of admissions. A potentially avoided emergency department visit was associated with mortality within 30 days (22.9%), X^2^ (1)= 9.82, *p* =.002, and urgent admission to the rapid access clinic (31.5%), *p* <.001, OR = 22.6 (95% CI: 7.63, 66.87). There were more planned (67.5%) than urgent admissions. Mortality within 30 days of presentation to the clinic was 24.6%, and significantly associated with male gender (31.3%), X^2^ (1) = 6.02, *p* =.014 and urgent admission (34.8%), X^2^ (1) = 6.7, *p* =.008.

**Conclusions:**

A newly established palliative care rapid access clinic addressed acute symptoms in a timely manner and may offer a valuable alternative to emergency department care, particularly for patients nearing the end of life. Further prospective research using control groups and validated patient outcome measures would provide more robust evidence about the clinic’s effectiveness in optimising end-of-life care and reducing the burden on our emergency departments.

## Introduction

Emergency department (ED) attendance for patients with palliative care needs who are acutely symptomatic may result from limited access to timely and appropriate outpatient palliative care or alternative urgent care [1, 2]. Emergency department visits are considered an indicator of suboptimal care for this patient cohort, as they can be poorly equipped to meet the complex needs of patients with palliative care needs [1, 2]. Visits to the ED can be distressing [3], and a high proportion of palliative patients report dissatisfaction with ED visits and would prefer alternative care settings, provided the quality of care is comparable [4–7]. However, this patient cohort may have more frequent ED visits in the last 30 days of life, and those presenting to ED in the last 2 weeks of life are often admitted to, and die in acute hospitals, further indicating poor quality of care [8].

Alternative models of care for patients with palliative care needs have existed in the form of Palliative Care Day Units (PCDU) since 1975 and are designed to enhance patient independence and quality of life through rehabilitation, symptom management and psychosocial support [9]. Typically, PCDU are nurse-led, have access to multidisciplinary teams, and encourage frequent visits. Some are staffed by volunteers, with funding largely derived from charitable contributions, and many lack regular access to a physician [9]. Socially driven models emphasise creative arts, complementary therapies, and respite care, while medical models concentrate on symptom assessment and other medical interventions [10, 11]. However, the distinction between these models is not well-documented, as both often provide similar services [10].

In Australia, research on PCDU is notably lacking and access to these units is more limited compared to other countries [12, 13]. It is hypothesised that PCDU might have a role in reducing ED visits [6], but supporting evidence is lacking and it is identified as a critical knowledge gap in PCDU research [14]. Rapid access clinics (RAC) have successfully addressed urgent patient needs for chest pain [15], radiation therapy [15, 16], and neurological disease [17]. However, in Australia, these models are rarely applied to palliative care, and no research evidence was found that describe this model of care in the palliative care context.

In 2020, the Sunshine Coast Health Specialist Palliative Care (SPC) service introduced a RAC to address patients’ unmet acute care needs by providing prompt, comprehensive, and time-critical care. This research aimed to better understand the characteristics and clinical outcomes of the patients attending the RAC, including whether an ED visit was potentially avoided, 30-day mortality, and whether the admission was planned or urgent. We used an exploratory, descriptive, retrospective cohort research design to address the aims of the study.

## Methods

### The Sunshine Coast Health rapid access clinic (RAC) model

The Sunshine Coast region of Queensland has a population of 459,912 people [18]. It is served by a large regional public health service with 5 public hospital and community services, including a SPC service comprised of community-based team, hospital in-reach team, and dedicated 18-bed inpatient ward. The RAC was located at one of the sub-acute public hospitals that included the inpatient unit and where the community outreach team was based. The RAC provided day-only services between the hours of 08:00 and 17:00 and initially operated two days per week. After the RAC was embedded and the model adequately tested, it was extended to 5 days a week, but this occurred after the data collection period. Limitations to further extension included lack of onsite ancillary services and restriction on staffing after usual business hours and on weekends.

The RAC was staffed by a medical officer and clinical nurse, with clinical governance provided by a senior medical officer in the SPC team. To facilitate rapid access, patients’ needs were initially triaged via phone. Patients were then reviewed that day in the RAC. The service aimed to facilitate a patient’s return to their usual residence after review. However, if this was deemed inappropriate, admission to an inpatient facility (inclusive of the co-located inpatient unit) was arranged. Referral to ED was arranged if the patient did not meet referral criteria or clinic capacity was exceeded.

There were two types of admissions: planned and urgent. Planned admissions were arranged for routine follow up after discharge from an inpatient unit, for patients deemed too complex to attend outpatient clinics, lack of a timely outpatient appointment, for blood transfusion or medication infusions. Urgent admissions to the RAC occurred if a SPC outreach team nurse, community general practitioner (GP) or community nursing service requested urgent review of feeding tubes, catheters, drains or indwelling vascular access lines, pain and other symptom exacerbation, or complex wound management.

The medical officer and nurse completed an initial assessment. Management plans were developed in consultation with the patients’ GP to ensure continuity of care as needs evolved and aligned with the patient’s home environment and available carer support. Complex management issues were addressed in consultation with the SPC senior medical officer. Resuscitation orders and advance care planning were routinely completed, with allied health support accessed from co-located services. Community-based nursing or domiciliary services were arranged for patients not already receiving support.

### Research design

This study analysed the electronic and paper-based patient records of palliative care patients who attended the RAC between 1 January 2020 and 30 December 2022. The study population was comprised of patients already known to the SPC service, aged 18 years or older, with a prognosis of less than 12 months and whose goals were focussed on symptom management, rather than curative intent such as chemotherapy or radiotherapy. The opening hours and lack of ancillary service availability such as onsite pathology, a pharmacy providing a full scope of services and limited radiology service availability narrowed the scope of available treatment. As a result, exclusion criteria needed to be established for treatment of patients with infections requiring immediate or intravenous antibiotics, medical conditions requiring lengthy or acute inpatient treatment, or individuals with significant behavioural disorders or delirium, except for terminal delirium.

Data from records of patients who attended the RAC were extracted from electronic records and collated from paper-based medical records by two of the authors (T.R. and M.M.) and included demographic and diagnostic information, reason for admission and date of death. Mortality at 30 days after discharge from the RAC was recorded as a clinical outcome. Clinical outcomes also included whether the admission was planned or urgent and whether the admission potentially avoided an ED visit.

There is a lack of standardised criteria for determining whether an ED visit was avoidable [1]. As a result, this was a subjective judgement, although pre-determined criteria were established. A potentially avoided ED visit was defined as an admission following an assessment by the second author (TR) confirming that the patient’s symptoms or general decline were significant enough to warrant direct admission to the SPC inpatient unit, medical ward or private hospital, and could have resulted from either a planned or urgent admission. In addition to this, a potentially avoided ED visit required that the care requirements could not have been delivered in an ambulatory care setting. To enhance internal validity, a random sample of 30% of patient notes was independently reviewed by another medical research team member (MM). In cases of uncertainty, consensus was reached through discussion.

### Statistical methods

Deidentified data were collated in Microsoft Excel, and subsequently imported into *R* version 4.1.0 for analysis. Quantitative data were analysed using both descriptive and inferential statistics. Demographic information was analysed using frequencies, percentages, and measures of central tendency (e.g., mean).

Statistical analysis examined differences between variables for patients who had a planned or urgent admission, whether an ED visit was potentially avoided, and mortality within 30 days of the RAC admission. The age variable was dichotomised in two groups for analysis: patient age below the mean age (rounded to 70 years) and those greater than or equal to the mean age. Other variables analysed included gender, and reason for admission to the RAC, using the chi squared test, a non-parametric test for non-normally distributed data. Fisher’s exact test was used with a two-sided alternative hypothesis to assess associations between the type of presentation and whether an ED visit was potentially avoided. Confidence interval was set at 95%.

## Results

There were 283 admissions to the RAC for 172 unique patients during the study period, with slightly more male (52.3%) than female patients. The mean age was 69 years, with a range from 29 to 98 years. Most patients (94.2%) had a terminal cancer diagnosis (see Table 1).

**Table 1:** Diagnoses of patients admitted to the RAC

Primary diagnosis	*n*
Genitourinary cancer	30 (17.4%)
Colorectal cancer	27 (15.7%)
Hepatobiliary cancer	22 (12.9%)
Lung cancer	21 (12.2%)
Upper gastrointestinal cancer	16 (9.3%)
Head and neck cancer	13 (7.6%)
Non-malignant	10 (5.8%)
Other malignant	8 (4.7%)
Haematological cancer	7 (4.1%)
Breast cancer	7 (4.1%)
Cutaneous cancer	6 (3.5%)
Central nervous system cancer	5 (2.9%)
**Total**	**172**

Admissions were either planned or urgent, and either admission type could have resulted in ED avoidance. There were more (67.5%) planned admissions, with an almost equal gender distribution of females (50.8%) and males (49.2%). Most planned admissions were for patients over 70 years of age (62.3%). The most common reason was for blood or medication transfusions (42.4%) given for symptomatic management of anaemia and when treatment aligned with patient goals relating to quality of life.

Nearly a third of admissions (32.5%) were urgent, with a higher proportion of males (59.8%) requiring urgent admission. The primary reason for urgent admissions was symptom or wound review (92.4%) (see Table 2).

**Table 2:** Demographics, reason for admission, and death within 30 days for planned and urgent admissions

	Planned	Urgent	Total
	*n* = 191 (67.5%)	*n* = 92 (32.5%)	*n* = 283
**Age group**			
< 70	72 (37.7%)	50 (54.3%)	122 (43.2%)
> 70	119 (62.3%)	42 (45.7%)	161 (56.8%)
**Gender**			
F	97 (50.8%)	37 (40.2%)	134 (47.7%)
M	94 (49.2%)	55 (59.8%)	149 (52.3%)
**Reason for admission**			
Blood/medication infusion	81 (42.4%)	0 (0%)	81 (28.4%)
Catheter/drain/PICC line	12 (6.3%)	7 (7.6%)	19 (6.7%)
Symptom/wound review	29 (15.2%)	85 (92.4%)	114 (40.7%)
Post discharge/RAC review	69 (36.1%)	0 (0%)	69 (24.2%)
**Died within 30 days**			
N	153 (80.1%)	60 (65.2%)	213 (75.4%)
Y	38 (19.9%)	32 (34.8%)	70 (24.6%)

Younger patients were significantly more likely to require urgent reviews (41.0% vs. 26.1%), X^2^ (1)= 6.534, *p* =.011. No significant association was found between gender and the need for an urgent review, X^2^ (1) = 2.504, *p* =.114. (see Table 3).

**Table 3:** Demographics associated with admission type

	Planned	urgent	*P*
	*n* = 191 (67.5%)	*n* = 92 (32.5%)	*n* = 283
**Age group**			
< 70	72 (59.0%)	50 (41.0%)	
> 70	119 (73.9%)	42 (26.1%)	0.011
**Gender**			
F	97 (72.4%)	37 (27.6%)	
M	94 (63.1%)	55 (36.9%)	0.114

Potentially avoided ED visits were recorded for 11.7% of the RAC admissions, with most (87.88%) for the urgent admissions, primarily by facilitating a direct admission to the SPC inpatient unit for care (51.5%) (see Table 4).

**Table 4:** Reasons ED visit was potentially avoided

	Planned	Urgent	Total
	*n* = 4 (12.12%)	*n* = 29 (87.88%)	*n* = 33
Admission to inpatient PCU	3 (9.1%)	17 (51.5%)	20 (60.6%)
Urgent intervention not possible as outpatient	0 (0%)	8 (24.2%)	8 (24.2%)
Admission to oncology ward	1 (3%)	2 (6.1%)	3 (9.1%)
Admission to private hospital	0 (0%)	2 (6.1%)	2 (6.1%)

Patients judged to have an ED visit avoided were significantly more likely to die within 30 days of discharge from the RAC (22.9% vs. 8.0%), X^2^ (1) = 9.82, *p* =.002. Urgent admissions were strongly associated with potentially avoided ED visits (31.5% vs. 2.1%), *P* <.001, OR = OR = 22.6 (95% CI: 7.63, 66.87) (see Table 5).

**Table 5:** Characteristics associated with potential avoidance of ED visit

*n* = 283	ED visit not avoided	ED visit potentially avoided	*P*
	*n* = 250 (88.34%)	*n* = 33 (11.66%)	
Age			
< 70	103 (84.5%)	19 (15.5%)	
> 70	147 (91.3%)	14 (8.7%)	0.095
Gender			
F	121 (90.3%)	13 (9.7%)	
M	129 (86.6%)	20 (13.4%)	0.371
Died within 30 days			
N	196 (92.0%)	17 (8.0%)	
Y	54 (77.1%)	16 (22.9%)	0.002
Type of Admission			
Planned	187 (97.8%)	4 (2.1%)	
Urgent	63 (68.5%)	29 (31.5%)	< 0.001, OR = 22.6

There were no significant associations between potentially avoided ED visit and age, X^2^ (1) = 2.79, *p* =.095, gender X^2^ (1) = 0.80, *p* =.371. or 30-day mortality X^2^ (1) = 0.95, *p* =.329. (see Table 6).

**Table 6:** Characteristics associated with a potentially avoided ED visit in the urgent cohort

	ED visit not avoided	ED visit potentially avoided	*P*
	*n* = 60 (75.95%)	*n* = 19 (24.05%)	*n* = 79
Age			
< 70	30 (64.6%)	17 (35.4%)	
> 70	30 (72.1%)	12 (27.9%)	0.591
Gender			
F	24 (65.8%)	13 (34.2%)	
M	36 (69.8%)	16 (30.2%)	0.839
Died within 30 days			
N	41 (72.4%)	16 (27.6%)	
Y	19 (60.6%)	13 (39.4%)	0.329

Mortality within 30 days of discharge from the RAC was 24.6%, with male patients significantly more likely to die within this timeframe (31.3% vs. 17.6%), X^2^ (1) = 6.02, *p* =.014. Urgent admissions were associated with a significantly higher mortality rate (34.8% vs. 19.9%,) X^2^ (1) = 6.7, *p* =.008. There was no significant association between age and 30-day mortality, X^2^ (1) = 0.13, *p* =.72 (see Table 7).

**Table 7:** Characteristics associated with 30-day mortality

	Did not die within 30 days	Died within 30 days	*P*
	*n* = 213 (75.3%)	*n* = 70 (24.7%)	
Gender			
F	112 (82.4%)	24 (17.6%)	
M	101 (68.7%)	46 (31.3%)	0.014
Type of Admission			
Planned	153 (80.1%)	38 (19.9%)	
Urgent	60 (65.2%)	32 (34.8%)	0.008
Age			
< 70	91 (74%)	32 (26%)	
> 70	122 (76.3%)	38 (23.7%)	0.720

## Discussion

This research aimed to describe the RAC service, the admitted patient characteristics and clinical outcomes. We found that a newly established SPC RAC provided both planned and urgent services to patients known to the palliative care service. We judged that the RAC prevented 33 ED visits, while still providing high-quality urgent care to people approaching the end of life. Urgent admissions to the RAC were associated with significantly higher mortality, male gender, and the patient cohort under 70 years of age. Mortality at a point 30 days after the RAC admission was 24.6%.

Positive outcomes have been reported for RAC clinics for other medical specialities. In a study evaluating a rapid access chest pain clinic, the authors described a reduction in ED admissions from 73.9 to 46.5%, as well as a reduction in bed days and fewer invasive tests [15]. Similarly, an urgent care clinic for patients with cancer resulted in 166 (41.5%) avoided ED visits for 400 patients triaged via telephone [19]. In a study of a RAC for neurology, the authors reported a reduction in wait times for an outpatient appointment, and requests for urgent consultations to the ED [17].

No evidence was found in the literature about RAC for specialist palliative care. Palliative care day units that implement medical, social or mixed approaches to care delivery have reported varying outcomes. Positive outcomes have been reported for distress from symptoms and functional abilities [20], although the same study found a decrease in quality of life. Higginson et al., (2010) found no difference in the use of hospital services after the commencement of a hospice day care program [21]. However, the heterogeneity of day care models makes comparison difficult.

The finding that the RAC potentially allowed patients to avoid an ED visit is important, however should be interpreted with caution. Prior to the RAC commencing, patients with similar presentations were typically advised to attend the ED, but it is uncertain whether all patients would have followed this advice. Nonetheless, despite a preference amongst palliative patients to avoid the ED whenever possible, the absence of an alternative care setting at the time would likely have left them with no choice but to seek urgent care through the ED [2, 5–7, 22].

The finding that 75.8% of cases where an ED presentation was likely avoided resulted in subsequent inpatient admission is notable. Historically, patients who were not deemed appropriate for direct ward admission but required urgent medical review for acute symptom exacerbation would have been directed to the ED. Applying the approach used by Delgado et al. (2015) which categorised unavoidable ED presentations as often involving sudden, severe symptom onset [1], and a high proportion (64%) requiring inpatient admission, would support the findings of this study. In addition, the relationship between ED avoidance and 30-day mortality may be more reflective of the underlying severity of the patient’s condition, rather than an effect of the intervention itself. The patients who died may also have had a more established relationship with the SPC team and felt comfortable contacting the SPC team for immediate care.

One of the most notable considerations for this research is the clear definition as to what constitutes a prevented ED admission. A patient or clinician in an outpatient clinic may be at risk of selection and confounding bias, such as satisfaction with the service impacting judgement about whether the patient may have attended an ED if the alternative was not available [23]. This could be addressed by using a comparison group as part of a controlled trial. There is some evidence from the literature about which ED admissions were avoidable, including visits for conditions that were not urgent, could have been managed in an outpatient primary care clinic, via phone review such as medication prescription [1] or short time frame spent in ED [24]. These are often established using retrospective analysis of medical records by medical officers or other health professionals using pre-defined criteria. This approach can be difficult, as, for example, a primary diagnosis of abdominal pain may be considered non-avoidable, but an underlying constipation considered potentially avoidable [1]. The decision to attend ED or not may be influenced by factors such as the presence of an advance directive, the lack of availability of primary care services such as a GP, or patient preference [1]. Nonetheless, this represents a reproducible definition of avoidable ED visits for future research, particularly in a more well-established RAC.

The primary means of triaging patients to present to the RAC was via telephone, which may not have been the most effective approach. Triage in palliative care is inherently complex, with no widely accepted or standardised tools currently available [25, 26]. Palliative patients often present with diverse and complicated symptoms that are best assessed through face-to-face consultations to accurately determine their disposition [27–29]. However, this was the best method available for time-critical decisions when patients telephoned the service experiencing high levels of distress. Inconsistent approaches to triage, or inaccurate triaging processes may have led to patients being inappropriately directed to ED.

To mitigate the shortcomings of telephone triage, experienced palliative care nurses conducted the screening and discussed the patient with the senior medical officer from SPC on duty. Further refinement of the triage pathway could help facilitate the most appropriate care pathways for patients, including direct ward admission where feasible. Incorporating tools such as the Australia-modified Karnofsky Performance Status (AKPS) [30], Palliative Care Problem Severity Score (PSS) [31], and Palliative Care Outcome Collaboration (PCOC) [32], has been shown to improve telephone triage accuracy [33], but their use was poorly documented in patients’ records. Further research into the use of these tools could provide valuable information about the appropriateness of telephone models of triage, and whether alternative models such as telehealth may be more efficacious.

There were operational challenges for the RAC model of care. Policies and procedures needed to be developed to ensure consistent care in the context of staffing changes [34], and access established to pathology and other services that were not co-located. Awareness of the clinic was limited as it was a new service with restricted days and hours due to funding, and as ongoing funding had not been confirmed, it was difficult to widely promote. An initial funding contribution was made by the Country to Coast Primary Health Network for a part-time medical officer, with the service needing to demonstrate a sustainable service to justify ongoing funding. The establishment of a model of care was challenging due to limited information about potential demand, consistent staffing availability, ensuring sufficient funding for justifying the service on an ongoing basis [35], and not overscheduling in a way that may compromise patient safety.

The location and operating hours of the RAC also constrained the types of treatment that could be delivered. The service was in a sub-acute hospital that did not have access to a pharmacy with a full range of medications, nor onsite pathology. Radiology was available but the scope of services and hours of operation were limited. In practice, for example, patients with infections could be prescribed oral antibiotics for collection at a community pharmacy but were unable to commence immediate intravenous antibiotics requiring multi-day treatment. Blood transfusions were necessarily planned as blood products needed to be ordered from an offsite blood bank and commenced early in the day.

The RAC’s ability to facilitate rapid transfers to appropriate SPC services for end-of-life care, bypassing the ED, was a key advantage. Care from a SPC service has been shown to produce statistically and clinically significant results in quality of life and emotional well-being, particularly when a multidisciplinary approach is used [36]. The RAC allowed a comprehensive assessment and management plan to be developed in collaboration with the patient, their carers and their GP with whom patient care is ideally shared [37]. This provided palliative care clinicians with an opportunity to urgently review treatment plans, care strategies, and community supports. Increasing support for carers may reduce their distress, improve well-being [38], and strengthen their ability to provide care for the patient at home [39].

The next steps in confirming the relationship between the RAC model of care and clinical outcomes is to conduct larger, prospective studies with control groups to validate the results and further investigate factors associated with ED avoidance and clinical outcomes. Establishing a priori criteria for classifying ED avoidance is essential to accurate capture this outcome. Patient Reported Experience Measures (PREMs) and Patient Reported Outcome Measures (PROMs) could be incorporated into clinical assessments to provide more robust evidence of the RAC effectiveness in improving symptom management and reducing aggressive end-of-life healthcare utilisation, particularly during the final 30 days of life.

### Limitations and strengths

The retrospective, observational nature of the medical record analysis meant that there were limitations. Some data were missing, and data analysis was dependent upon the quality of documentation [40]. This study was exploratory in nature, and aimed to better understand the characteristics and clinical outcomes of the patients attending the RAC. We acknowledge the limitations of the data analysis, and the lack of multivariate analysis leaves the variables open to confounding. Future research should include such methods to confirm these results and provide a richer analysis of patient outcomes. The study researchers were not blinded to the research objectives, making it challenging to eliminate bias [41]. Being a pilot study of a new clinic in one setting, the findings may not be generalisable to other settings [40].

Despite these limitations, this study offers valuable data about a novel, person-centred service. It demonstrated the feasibility of a RAC model for palliative care and how such a service has the potential to reduce ED visits for individuals with acute palliative care needs. Opportunities to strengthen knowledge about the outcomes of RAC through further research in the SPC discipline have been identified.

## Conclusion

A SPC RAC can play a valuable role in meeting patients’ urgent care needs outside of acute hospital settings. Our experience suggested that RACs may have the potential to reduce the need for people with palliative care needs to present to an ED with acute symptomatology, and support people who are approaching the end of life. Further prospective research utilising control groups is essential for evaluating this novel model of care. Future studies including more robust methods for determining ED avoidance, evaluating the efficacy of triage methods and validated patient outcome measures to further evaluate the impacts of SPC RAC would be a valuable addition to the existing research.

## Data Availability

The datasets used and/or analysed during the current study are available from the corresponding author on reasonable request.

## Abbreviations

ED: Emergency department
GP: General practitioner
RAC: Rapid access clinic
SPC: Specialist palliative care
